# Construction Accidents in Spain: Implications for an Aging Workforce

**DOI:** 10.1155/2022/9952118

**Published:** 2022-06-02

**Authors:** Ignacio Fontaneda, Miguel A. Camino López, Oscar J. González Alcántara, Birgit A. Greiner

**Affiliations:** ^1^Industrial Engineering, University of Burgos, Burgos, Spain; ^2^Economic Science, University of Burgos, Burgos, Spain; ^3^School of Public Health, University College Cork, Ireland

## Abstract

Construction workers are getting older. In the European Union, the percentage of workers over 50 grew from 24.7% in 2011 to 31.5% in 2018, in Spain from 20.4% to 31.2%. *Objective*. Identify trends and detailed patterns of accidents of older construction workers compared to other age groups. *Data and Method*. We analyzed construction accidents in Spain from 2011 to 2018 (*N* = 455,491). The number of accidents and lost working days (LWD) were broken down by occupation, seniority, company size, temporal variables (weekday, hour), trigger, and body part injured and compared for different age groups. *Results*. Although older worker had fewer accidents, the consequences of accidents were more serious. Those over 50 years had 84% more lost working days (LWD) than those under 24 years, 48% more than those between 25 and 39 years, and 21% more than those between 40 and 49 years. (1) Occupation: the percentage of accidents grew with age for supervisors, lorry drivers, and bricklayers. (2) Seniority: the least experienced (less than 6 months) and the most experienced (more than 6 years) had the most LWD. (3) Company size: there are 24.5% of accidents in companies of less than four workers. (4) Trigger: older workers suffered more falls, both from height and at the same level. (5) Time: the percentage of accidents in those over 50 was higher on Thursdays and Fridays, in the afternoons from 4 to 7 p.m., and after four hours of work. (6) Injury: this shows the longest absences for shoulder injuries for those over 50 years, with an average of 70 LWD.

## 1. Introduction

Worldwide, life expectancy has increased between 1950 and 2017, from 48.1 years to 70.5 years for men and from 52.9 years to 75.6 years for women [1]. This equates to a four-month increase in life expectancy each year. With fertility decline, increase of longevity, and progression of large-sized cohorts (baby boomers in some countries) to the older ages, population aging is a dominant demographic phenomenon [2].

Western Europe has one of the oldest populations, with a population over 65 years of 17% in 2010 that is predicted to increase to 30% by 2060 [3].

The population increase of the elderly group challenges the sustainability of the pension systems across Europe and the rest of the developed world [4]. An increase in older workers' participation in the labor market is necessary to sustain the pension systems [5, 6]. To address this challenge, many European countries are increasing the official age for retirement up to 65 years and even further [7–9] . The normal retirement age of men will increase in 20 out of 36 OECD countries by an average of 3.5 years based on current legislation [9]. Many countries are establishing incentives to retain older workers in the workforce and thus alleviate the challenges of population aging [10]. The mix of younger and older people in the workforce is changing and will further change in the future.

According to the International Labor Organization (ILO), the percentage of workers over 65 years of age worldwide was 3.2% in 2000 and 3.7% in 2015 and is expected to be 5.3% in 2030. The percentage of workers over 55 years will grow worldwide; for those between 55 and 64, it was 7.8% in 2000 and 11% in 2015 and is expected to be 13.3% in 2030 [11].

In Europe, between 2000 and 2010, the share of workers over 55 rose in 25 of the then 27 EU Member States [12]. Between 2003 and 2018, those aged 55 and over increased from 12.1% to 19.7%, those aged 55-64 increased by 45.5%, and those who were aged 65 years or more increased by 52.1% [13].

The relationship between aging and occupational safety and health showed contradictory evidence. In a meta-analysis including studies from the last 30 years, Peng and Chan [14] calculated the pooled incidence rate of fatal accidents; for older workers, it was double than that of younger workers, but nonfatal accidents were slightly lower (5.8%) in older compared to younger workers. A review from Salminen [15] on fatal occupational injuries, 29 out of 45 studies, showed that young workers had a lower fatality rate than older workers. In another calculation, Jackson [16] established that older workers had approximately half as many injuries as younger workers, but in the event of an injury, it took them almost twice as long to recover. The recovery time was shown to be longer for older workers after an accident [17] with usually more severe injuries generally linked to higher injury costs associated with the accident [18]. Age is an important factor in injury involvement [19].

The severity of the accident and lost working days may vary by work activity [20], with the occupation being an important moderator in the relationship between aging and safety [14, 21].

Aging is associated not only with a general decline in physical and cognitive functioning [14, 22, 23] but also with increases in experience [24, 25]. Work in construction is among the most physically demanding [18], with frequent lifting, carrying heavy materials and static work [23], and repetitive manual tasks in awkward and cramped positions [26]. Physical activities in construction may be a challenge particularly for older workers [27–29].

In the USA, the proportion of construction workers aged 55 and older increased from 12% to more than 20% between 1985 and 2015 [27]. In the European Union, between 2011 and 2018, the percentage of construction workers over 50 years rose from 24.7% to 31.5% [30], in the UK rose from 26.9% in 2009 to 32.3% in 2018 [31], and in Spain rose from 17.5% in 2008 to 29.8% in 2018 [32].

Despite the challenges of an aging workforce in construction, there is little knowledge about how this will affect accident leave rates [33], and there are only few studies on this topic [6], with a focus on injuries due to falls and injuries leading to musculoskeletal disorders [18]. A more detailed analysis is lacking to develop more specific preventive measures which consider the respective risks associated with age [7, 34], the occupation, and the type of accident [35]. They would be also necessary to identify trends and specific intervention methods [18, 36].

The objective of this study is to identify patterns of accidents of older construction workers compared to other age groups in the Spanish construction sector, over the period 2011-2018.

To do this, we will test the hypotheses:

- H1a: The incidence rate was the lowest for the oldest and increased as age decreased
- H1b: Lost working days due to an accident increased as age increased
- H2: The age of the injured workers is not independent of the rest of the variables under study (older people have more accidents in different occupations, in relation with seniority, in large enterprises, with different triggers and in relation with time (day and hour), and with different parts of the body affected)
- H3: Older workers injured have a longer duration of sick leave (lost working days—LWD) in all cases (occupation; seniority; company size; deviation-trigger; day, hour of the day and hour of work; and injured body part)

## 2. Materials and Methods

### 2.1. Data Collection

This is a secondary data analysis study using official accident data. In Spain, any bodily injury sustained by a worker that arises out of or in connection with work is defined as an occupational accident [37]. All occupational accidents that involve any lost working days (LWD) must be submitted electronically via an accident report form. The accident reporting systems offer a significant financial compensation for the victim when an accident is reported.

The data for this analysis were collected from the official Workplace Incident Notification Forms, held on file at the Ministry of Labor, Migrations and Social Security in Spain. We selected all accidents, resulting in sick leave of more than one day, from the construction sector (NACE code = F) in Spain, over the period 2011 through 2018. Relapses and commuting accidents (travelling to and from work) were excluded from the study.

Table 1 shows accidents by year. The total number of accidents in the construction sector under analysis was 455,491. Of these, workers over 50 years old suffered 95,657 accidents in the period studied.

The large number of accidents empowers our ability to detect small but statistically significant differences.

### 2.2. Variables Analyzed

The official Workplace Incident Notification Forms gather information about the following [38]:

- Worker: occupation, age, sex, nationality, employment status, and seniority
- Company: size, economic activity (NACE code), and geographic location
- Temporal and spatial information: date, day of the week, day (hour) and work shift (hour), and workplace
- Sequence of events: specific physical activity and associated material agent and trigger (“cause of the accident”—deviation and associated material agent)
- Injury: severity of the accident, type of injury, and injured body part
- End of the injury leave: the number of lost working days (LWD) and the cause of the discharge at the end of the injury leave

### 2.3. Study Design

To compare accidents at different ages, we have grouped them into four strata: under 24 (young), from 25 to 39 (middle-young age), from 40 to 49 (middle-old), and over 50 years (old).

**Figure 1:**
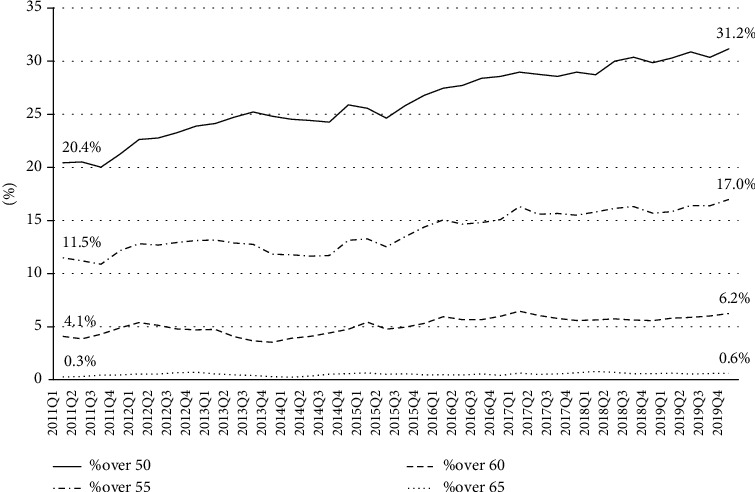
Workers in construction by age, Spain, 2011-2018 [30].

The group over 50 years represented between 20% (third quarter of 2011) and 31.2% (fourth quarter of 2019) of workers in the construction sector in Spain (Figure 1), an average of 25.7% from the first quarter of 2011 to the fourth quarter of 2018 (period under analysis). The groups over 55 years represented an average of 13.6%, over 60 years an average of 5%, and over 65 an average of 0.5% of all construction workers (Figure 1).

We analyze the differences between the age groups in number of accidents and average lost working days (LWD) due to the accident together with other reported variables to establish the differences in accident patterns and in their severity (measured by the number LWD).

### 2.4. Statistical Analysis

With respect to the number of accidents, using contingency tables, we tested the hypothesis of independence of age with each variable (occupation, seniority, company size, etc.) with the chi-squared statistic (*χ*^2^). If the significance level (*p* value) was less than 0.05, then we can affirm there is a difference in the number of accidents according to the age group for the analyzed variable.

If the different values of each variable do not influence the number of accidents at different ages, then the percentage of accidents in the different age groups will remain uniform for the different values of the variable (ex. the group over 50 years will suffer 21% of accidents). If the differences in percentage are statistically significant, those values of the variable will influence the accident rate at different ages. The percentage of a cell with a value significantly greater (statistically) than that corresponding to the respective age group (percentage of the column) is marked with an ∗ and shadowed. This way, we highlighted the values of the variable that increases the expected number of accidents, where preventive measures could be taken.

We calculated, for each variable under analysis, the average of lost working days (LWD) due to an accident by age group and performed the Kruskal-Wallis *h* test to determine if the LWD rank differences were statistically significant for each variable (sig.<0.05). We used a nonparametric test and the Kruskal-Wallis *h* test, instead of the parametric test of ANOVA (one-way analyses of variance) because LWD were not normally distributed and the variation of LWD was not homogeneous in the groups.

We conducted all analyses using SPSS v23 software.

## 3. Results

**Figure 2:**
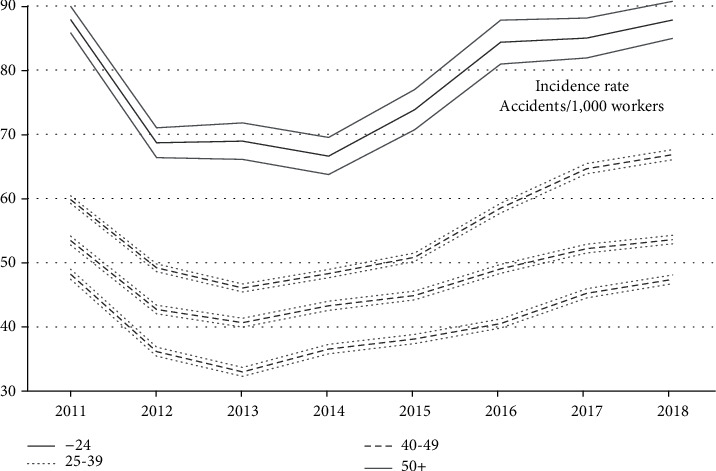
Incidence rate in construction by age (with 95% confidence intervals), Spain, 2011-2018.

The incidence rate was the lowest for the oldest (over 50) and increased as age decreased. Those under 24 years of age had the highest incidence rate every year (Figure 2).

The incidence rate (accidents/1,000 workers) for construction workers over 50 years ranged between 48.2 in 2011 and 47.4 in 2018 with a minimum of 33 in 2013. This incidence rate was lower for older workers compared to the other age groups. The incidence rate was especially high for those under the age of 24, reaching 87.9 in 2011 and 87.8 in 2018 (Figure 2).

**Figure 3:**
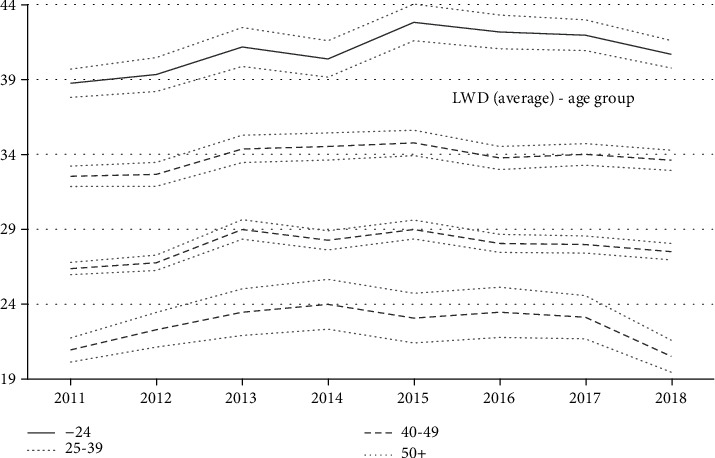
Average lost working days (LWD) in construction by age (with 95% confidence intervals), Spain, 2011-2018.

However, in the event of an accident, these seemed to be more serious for the elderly as the average lost working days (LWD), due to an accident, was higher for older worker groups for every year (Figure 3). From 2011 to 2018, the average lost working days was 22.2 for those under 24 years, 27.7 for those between 25 and 39 years, 33.7 for those between 40 and 49, and 40.9 for those over 50 years. When they had an accident, those over 50 years had 84% more LWD than those under 24 years, 48% more than those between 25 and 39 years, and 21% more than those between 40 and 49 years.

In addition, the fatality rate (fatal accidents/100,000 workers) was higher for older workers, with an average, between 2011 and 2018, of 11.7 for construction workers over 50 years, 8 for those between 40 and 49, and 4.7 for those between 25 and 39.

### 3.1. Accidents and Average Lost Working Days (LWD) by Occupational, Individual, and Temporal Variables

Table 2 provides an in-depth analysis of accidents and lost working days broken down by occupation. In the following occupation, the older age group was particularly affected by accidents (compared to 21% of all accidents suffered by those over 50): mining, manufacturing, and construction supervisors (38.5%), heavy truck and lorry drivers (31.5%), and bricklayers and stonemasons (27%).

The average lost working days, due to an accident, was different between the different occupations. In all occupations, the lost working days increased significantly with age in the event of an accident (K-W test, Table 2), with particular high LWDs in the over 50 years old age group for mobile plant operators (50.6 days), painters (46.8 days), and heavy truck and lorry drivers (44.5 days).

The accidents suffered by workers with less than 3 months of seniority had the worst consequences (the highest average in LWD), followed by the lost working days per accident suffered by the most experienced (more than 6 years). Regardless of seniority in the position, the days lost due to the accident increased with age (K-W test), this increase was greater for workers with less than 3 months of experience (Table 3).

A high number of accidents occurred in microenterprises, 24.5% of accidents in companies of less than 4 workers and 17.4% in companies between 5 and 9 workers (Table 4). These companies had a higher proportion of accidents of younger workers, and the average LWD was higher for all age groups (K-W test, Table 4). Nearly 6.4 million working days have been lost due to an accident in construction companies with less than nine workers, nearly eight hundred thousand a year. Independently of company size, LWDs increased by age.

Older workers (over 50) suffer more falls, both from height (24.6%) and at the same level (25.9%) with respect to the total accidents in that age group (21%), while for younger workers, the loss of the control of equipment or materials, electrical problems, overflow, and breaks of material was more prevalent in accidents (Table 5). The severity of accidents occurs due to falls at different levels, with an average of 62.1 LWDs, more than double the average number of days lost due to the rest of accidents (29.85) and the highest in all age groups (Table 5).

Workers over 50 were more likely to be injured at the end of the regular workweek, on Thursdays and Fridays. The percentage of accidents in the elderly tended to grow as the week progressed from Monday to Friday, from 20.4% on Mondays to 22.6% on Fridays. Older workers, over 50, also had more accidents than expected for their age group in the afternoon and with more work hours, after the first four hours of work (Table 6).

The average lost working days (LWD) was higher for the older on all weekdays and at any hour of the day (Table 6). Point out that the average LWD grows throughout the workweek, with more days lost on weekend than Friday accidents, more on Friday than Thursday, more on Thursday than Wednesday, more on Wednesday than Tuesday, and with the lowest average LWD on Mondays. The average number of days lost also grows after four hours of work.

As for the physical consequences, upper limb injuries accounted for 33.8% and lower limb injuries 28.4% of all injuries. Younger workers, below 39 years, suffered more injuries in the fingers, hands, wrists, ankles, and feet (in the most extreme part of the extremities of the body), while older ones, over 50 years, suffered more injuries in the arms, shoulders, legs, and hips. The back accounts for 18.5% of all injuries (Table 7). The highest average of LWD is due to injuries of multiple parts, shoulder and humerus joints and hip and hip joint.

Recovery after leave was present in most of cases (at least 92.3% of the accidents). However, in the case of older workers, over 50 years, the probability of ending in legal incapacity for work or that the accident had fatal consequences was higher. Of the accidents of workers over 50 years, 1.2% ended in a legal incapacity for work, ten times the percentage of the youngest group (less than 24 years) with only 0.12% of their accidents ending in a legal incapacity for work and nearly four times of the group from 25 to 39 years (0.31%) and twice the percentage (0.57%) of the group from 40 to 49 years (Table 8). There is usually a long period of lost working days before the declaration of legal incapacity.

## 4. Discussion

Although in construction, young workers tend to have more accidents (Figure 2), older workers had more lost working days when they had an accident (Figure 3). Incident rates decreased significantly for all worker age groups, between 2011 and 2013. This decrease is probably due to the economic crisis of 2008, a crisis that lasted in Spain. During economic crises, there is a sort of “natural selection” where the best adapted (the best workers) tend to remain in the workplace, reducing the probability of having an accident [39]. Young workers have a higher incidence rate every year, despite year-on-year variations.

The findings that older workers are less likely to have an accident and more likely to suffer severe accidents are in line with the international accident research in construction [29, 40] and other industries [24, 34, 41]. However, our study also allowed a detailed analysis of occupational, temporal, and individual factors in this relationship. The trend of lost working days per accident increased with age, and this association was consistent in all occupational groups, for all company sizes, all causes of accidents, and injured body part.

Safety problems of older workers may well be restricted to activities that are specifically “age-impaired” [42]. Some occupations had a lower percentage of accidents in the older groups (Table 2); as electricians and plumbers, older workers seem to be more careful about the risks in these occupations. Their grown experience [24, 25] as well as company measures that allow them to work in less strenuous activities may explain these lower percentages. In other occupations (Table 2), like for bricklayers (27% of accidents when 21% is expected for workers over 50 years) or concrete placers (38.5% of accidents when 21% is expected for workers over 50 years), overexertion plays an important role and may lead to the increase in the percentage of accident for older workers. Physical strength and agility seem to be important in accidents involving the elderly as aging is associated with a general decline in physical and cognitive functioning [14, 22, 23]. Training on load handling is recommended and the prevention of overstraining, along with specific routines for staying fit and with more frequent breaks, for older workers, especially for bricklayers and concrete placers. Companies have a responsibility to adapt the workplace to the changing capabilities of the workers as they age. Some occupations are challenging, as the worker gets older, particularly physically demanding occupations [43].

The least experienced (less than 3 months—27.0% of the accidents) and the most experienced (more than 6 years—21.1% of the accidents) were those who suffer more accidents and a higher average of LWD in the case of an accident (Table 3). Preventive and training measures should focus on the beginning, especially for those who start older and take longer to adapt, so that they know the risks better. For those with more experience, overconfidence seems to be the cause of part of the accidents, hence the importance of establishing reminders of the risks of the workplace.

The most serious accidents occurred in microenterprises of less than nine workers, measured in the number of lost working days (6,339,716 LWD—43.8% of the total lost working days) and 41.9% of accidents (190,673). The improvement of working conditions and preventive measures in these microenterprises should be the focus of the administration in the construction sector.

Older workers should be especially careful with falls, as they have a higher percentage of falls (Table 5). Falls at different levels are the accidents with the worst consequences and the most LWD. More research is needed to clarify why older workers suffer more falls than younger ones.

Regarding temporal variables (Table 6), older workers had more accidents at the end of the regular workweek (Thursday and Friday). The percentage of accidents of older workers grows along the weekdays from 20.4% on Monday to 21.6% on Thursdays and 22.6% on Fridays. The percentage of accidents in the elderly (over 50) also increases as the day progresses (Table 6); they suffer the 20.8% of the accidents in the morning, the 21.2% during lunchtime, and 22.2% in the afternoon (between 4 and 7 p.m.). Older people also suffer more accidents after four hours of work. They suffer 20.6% of accidents in the first four hours of work and 21.6% when they have already worked more than four hours.

Further research is needed to analyze the causes of this higher percentage of accidents in the elderly as the week progresses, when the day progresses, and at the end of the work shift. We believe (in our opinion) that fatigue could play a role in these trends. If this hypothesis is confirmed, breaks and shorter working days-weeks could be proposed for the elderly.

Regarding the consequences of the accident, older workers tended to injure their joints, hip, shoulder, arm, and leg (Table 7). Joints, shoulder, and hip injuries accounted for the most lost working days. Gibb et al. [29] proposed reducing extreme joint movement (keeping motion within acceptable range), reducing excessive force (using mechanical aides), and reducing highly repetitive tasks (use of power tools) for reducing these types of accidents. LWD data for the different injured body parts can help to forecast the length of absence from work for different injured body parts by age group. We found the longest absences for shoulder injuries for those over 50 years, with an average of 70 lost working days.

As older workers had more fatal accidents than younger ones, an increase of fatal accidents in the construction industry can be expected at national level in Spain as the fatality rate will grow with an aging working population in this industry (Table 8) if measures to improve health and safety at work, especially for older workers, are not applied.

As retirement age approaches, if they have an accident, older workers are more likely to leave work [44]. Probably, at the end of their career, it is more effective to claim permanent disability or early retirement than continue to work, especially if full recovery is long.

The maintenance of older people's health and safety allows their contribution to the economy, rather than being disability recipients [45]. Companies can facilitate their contribution assigning them positions that take advantage of their experience, as trainers of the youngest, safety personnel or in less physically strenuous activities. To promote engagement and motivation, and keep working old workers working, companies should prevent age discrimination and procure organizational support for the elderly [46].

It is the responsibility for the companies to establish the organization of work, and it is the responsibility of the administration to make the necessary legal modifications for a progressive retirement.

### 4.1. Limitations

This study may not be generalizable to other countries. The study only analyzes accidents suffered by construction workers in Spain. In other countries, they used more modular construction procedures, while in Spain, construction continues in the traditional way, developing most of the work on site. A cross-national study analyzing accidents according to age in different countries would be necessary to confirm the differences found for older workers.

We are not able to calculate the incidence rate for different occupations, seniority, or company size, as we do not have data on the number of workers disaggregated according to these variables. We have only been able to calculate incidence rates for the entire sector. This limitation may introduce biases in the conclusions regarding these variables, since older workers may be more numerous in certain occupations, tend to have greater seniority, and may work in larger companies. We expect the bias to be smaller for time variables, assuming that most workers work full time.

The variables analyzed could be interdependent. For example, there may be interdependencies between the deviation (trigger of the accident), the day of the week, and the part of the body injured. Certain injuries may be more frequent on different days and due to different deviations. Multivariate statistical models need to be built to account for these interdependencies.

The data is anonymized, and we are not able to examine if some individuals, over time, are involved in multiple incidents, which prevents a longitudinal study. This limitation in the data makes it difficult to establish statistically cause-effect relationships.

### 4.2. Conclusions

The aging of the workforce, in almost all countries, has several implications for health, safety, and injury prevention.

Some occupations have a higher risk of accidents when workers age. Those over 50 have a higher percentage of accidents among supervisors, heavy truck-lorry drivers, and bricklayers.

The accumulated fatigue seems to influence an increase in the accident rate for workers over 50 (growing percentage of accidents as the week, the day, and the hours of work progress). We recommend reducing the working time and adequate breaks for the elderly, with shorter work shifts, especially at the end of the week (Thursdays and Fridays).

Our results show that there are more accidents, and they are more serious when starting in a new job (in the first three months) or with high seniority (more than six years of experience). Design of specific induction training at the beginning and prevention of overconfidence and complacency when seniority increases would be needed.

Jobs need to be adapted to the changing capabilities of workers as they age. Preventive measures should be taken for falls and overexertion. It is recommended especially for older workers to reduce extreme joint movement, excessive force, and highly repetitive tasks.

## Figures and Tables

**Table 1:** Number of accidents with lost working days by year in construction (Spain), 2011-2018.

2011	2012	2013	2014	2015	2016	2017	2018	Total
80,120	52,299	43,111	44,065	49,617	54,576	62,203	69,500	455,491

**Table 2:** Accidents and LWD by occupation and age in construction (Spain), 2011-2018.

Chi square = 14567.153423	Accident number	% of the accidents				LWD (average)					K-W test
Sig. (*p* value) < 0.01		-24	25-39	40-49	+50	-24	25-39	40-49	+50	Total	Sig.
Mining, manufacturing, and construction supervisors	2,867	0.5%	24.5%	36.6%^∗^	38.5%^∗^	12.8	32.4	37.9	43.6	38.7	Sig.<0.001
Concrete placers, concrete finishers, and related workers	30,451	3.3%	39.9%	34.8%^∗^	22.0%^∗^	22.6	28.6	35.9	42.0	33.9	Sig.<0.001
Bricklayers, stonemasons, stone cutters, splitters, and carvers	98,283	3.3%	35.8%	33.9%^∗^	27.0%^∗^	22.1	28.6	34.3	41.3	33.7	Sig.<0.001
Carpenters and joiners	5,220	5.8%^∗^	43.7%	29.5%	20.9%	21.3	26.3	31.4	39.5	30.3	Sig.<0.001
Building frame and related trade workers not elsewhere classified	41,403	3.8%	42.4%	32.3%^∗^	21.6%^∗^	21.4	28.2	33.5	40.4	32.3	Sig.<0.001
Plasterers	6,597	4.3%	48.1%^∗^	30.8%	16.8%	23.1	28.4	34.1	38.2	31.6	Sig.<0.001
Plumbers and pipe fitters	18,745	5.4%	48.7%^∗^	27.5%	18.4%	20.5	25.2	29.6	36.7	28.3	Sig.<0.001
Painters: spray painters and varnishers and related workers	10,190	4.3%	41.7%	31.9%^∗^	22.1%^∗^	22.9	30.7	38.6	46.8	36.4	Sig.<0.001
Floor layers and tile setters	2,871	4.3%	43.6%	32.0%	20.1%	23.2	27.0	34.1	38.7	31.5	Sig.<0.001
Air conditioning and refrigeration mechanics	7,658	5.7%^∗^	52.9%^∗^	26.1%	15.3%	24.2	25.4	30.0	38.0	28.4	Sig.<0.001
Roofers, insulation workers, glaziers, and building structure cleaners	28,414	4.2%	44.0%^∗^	31.3%	20.4%	24.6	27.6	33.4	40.9	32.0	Sig.<0.001
Sheet and structural metal workers, moulders and welders, and related workers	18,929	5.5%^∗^	48.0%^∗^	29.7%	16.8%	22.8	26.3	31.2	38.2	29.6	Sig.<0.001
Machinery mechanics and repairers	4,729	3.6%	44.2%	30.1%	22.0%	21.9	25.2	31.1	38.2	29.7	Sig.<0.001
Building and related electricians	26,977	5.7%^∗^	48.6%^∗^	27.6%	18.2%	21.5	27.2	32.2	41.7	30.9	Sig.<0.001
Electrical mechanics and fitters and electrical line installers and repairers	8,626	4.7%	48.6%^∗^	28.8%	17.9%	21.3	27.5	32.8	39.9	30.9	Sig.<0.001
Electronics and telecommunications installers and repairers	6,895	5.3%	51.0%^∗^	28.3%	15.5%	25.1	26.5	32.9	41.6	30.6	Sig.<0.001
Stationary plant machine operators	12,001	6.0%^∗^	50.8%^∗^	28.2%	14.9%	19.1	26.3	32.7	37.9	29.4	Sig.<0.001
Assemblers	5,703	6.7%^∗^	48.6%^∗^	29.4%	15.3%	18.7	27.3	31.6	39.7	29.9	Sig.<0.001
Mobile plant operators	7,687	2.3%	39.2%	33.5%^∗^	25.1%^∗^	23.1	31.8	37.9	50.6	38.4	Sig.<0.001
Heavy truck and lorry drivers	6,676	1.1%	30.5%	36.9%^∗^	31.5%^∗^	21.7	32.0	38.8	44.5	38.3	Sig.<0.001
Mining and construction labourers	66,198	9.8%^∗^	46.7%^∗^	27.4%	16.1%	22.4	27.6	33.9	40.1	30.8	Sig.<0.001
Manufacturing labourers	5,947	15.1%^∗^	51.4%^∗^	22.6%	10.9%	22.2	25.9	32.9	39.2	28.4	Sig.<0.001
Others	32,424	4.9%	41.6%	31.3%	22.2%^∗^	22.5	27.4	32.5	39.7	31.5	Sig.<0.001
	455,491	5.2%	42.9%	30.9%	21.0%	22.2	27.7	33.7	40.9	32.0	

^∗^ Percentage above the expected for this age (statistically significant). Sig. K-W test < 0.001 for all occupations. Growing LWD with age.

**Table 3:** Accidents and LWD by seniority and age in construction (Spain), 2011-2018.

Chi square = 18093.956	Accidents number	% of the row				LWD (average)					K-W test
Sig. (*p* value) < 0.01		-24	25-39	40-49	+50	-24	25-39	40-49	+50	Total	Sig.
Less than 3 months	123,132	7.3%^∗^	44.6%^∗^	30.3%	17.8%	23.4	30.4	37.5	44.5	34.5	Sig.<0.001
3 to 5 months	56,349	7.2%^∗^	45.5%^∗^	29.9%	17.4%	21.0	26.5	32.6	41.1	30.5	Sig.<0.001
6 to 11 months	55,179	6.8%^∗^	46.1%^∗^	30.0%	17.2%	21.0	26.2	32.0	38.3	29.7	Sig.<0.001
1 to 2 years	49,531	6.2%^∗^	46.1%^∗^	29.7%	18.0%	21.3	26.0	31.4	37.8	29.5	Sig.<0.001
2 to 3 years	27,402	5.1%	46.6%^∗^	29.7%	18.6%	21.6	25.9	31.6	39.4	29.9	Sig.<0.001
3 to 6 years	47,829	3.8%	47.8%^∗^	29.6%	18.8%	22.7	26.4	31.8	39.3	30.3	Sig.<0.001
More than 6 years	96,069	0.4%	32.5%	34.5%^∗^	32.7%^∗^	25.5	28.1	33.3	40.7	34.0	Sig.<0.001
	455,491	5.2%	42.9%	30.9%	21.0%						

^∗^Percentage above the expected for this age (statistically significant). Sig. K-W test < 0.001 for all seniorities. Growing LWD with age.

**Table 4:** Accidents and LWD by company size and age in construction (Spain), 2011-2018.

Chi square = 736.038930	Accident number	% of the row				LWD (average)					K-W test	
Sig. (*p* value) < 0.01		-24	25-39	40-49	+50	-24	25-39	40-49	+50	Total	Sig.	LWD (total)
Microenterprises (4-)	111,424	6.3%^∗^	43.1%	29.8%	20.8%	23.9	29.6	36.9	43.8	34.4	Sig.<0.001	3,828,915
Microenterprises (5-9)	79,249	5.3%^∗^	43.5%^∗^	30.4%	20.8%	22.5	28.3	34.1	41.3	32.4	Sig.<0.001	2,570,801
Small enterprises (10-19)	86,260	4.9%	42.9%	31.3%^∗^	20.9%	21.2	26.9	32.4	40.5	31.2	Sig.<0.001	2,689,501
Small enterprises (20-49)	98,877	4.8%	43.1%	31.4%^∗^	20.7%	20.5	26.2	31.8	38.8	30.3	Sig.<0.001	2,997,448
Medium-sized enterprises (50-249)	70,833	4.2%	42.0%	31.9%^∗^	21.8%^∗^	22.2	27.1	33.0	39.7	31.5	Sig.<0.001	2,233,485
Large enterprises (250+)	8,848	2.8%	40.8%	32.8%^∗^	23.7%^∗^	16.7	27.4	32.1	37.8	31.1	Sig.<0.001	275,065
	455,491	5.2%	42.9%	30.9%	21.0%							

^∗^Percentage above the expected for this age (statistically significant). Sig. K-W test < 0.001 for all company sizes. Growing LWD with age.

**Table 5:** Accidents and LWD by deviation (trigger) and age in construction (Spain), 2011-2018.

Chi square = 1743.258572	Accident number	% of the row				LWD (average)					K-W test
Sig.<0.001		-24	25-39	40-49	+50	-24	25-39	40-49	+50	Total	Sig.
Without information	6,951	5.3%	43.7%	30.6%	20.5%	20.6	25.2	31.6	39.2	29.8	Sig.<0.001
Electrical problem, explosion, and fire	4,168	5.6%	48.2%^∗^	28.6%	17.6%	24.6	28.0	33.1	43.5	32.0	Sig.<0.001
Overflow, overturn, leak, spill, and emanation	14,562	5.7%^∗^	46.0%^∗^	30.7%	17.6%	11.4	12.6	14.6	18.2	14.1	Sig.<0.001
Break, pop, slip, drop, and collapse material agent	39,360	5.7%^∗^	43.6%^∗^	30.1%	20.7%	22.4	28.8	35.7	42.1	33.3	Sig.<0.001
Total or partial loss of control of work equipment or materials	81,438	6.1%^∗^	44.8%^∗^	29.6%	19.5%	22.7	26.8	32.1	37.6	30.2	Sig.<0.001
Falling people from height	30,994	4.3%	40.0%	31.2%	24.6%^∗^	38.2	53.3	66.2	75.3	62.1	Sig.<0.001
Falling people at same level	43,004	5.1%	38.3%	30.6%	25.9%^∗^	26.7	34.4	42.1	49.3	40.2	Sig.<0.001
Falling people without specification	3,128	5.1%	41.4%	31.3%	22.3%	20.9	27.6	36.3	45.4	34.0	Sig.<0.001
Body movement without added physical effort	87,608	4.9%	43.0%	31.5%^∗^	20.6%	21.6	26.1	31.3	36.0	29.6	Sig.<0.001
Body movement as a consequence of or with physical effort	134,216	4.7%	43.2%^∗^	31.8%^∗^	20.4%	18.7	23.6	27.8	34.6	26.9	Sig.<0.001
Surprise, fear, violence, aggression, threat, and presence	2,341	5.7%	44.5%	30.2%	19.7%	23.5	31.3	39.9	49.0	36.9	Sig.<0.001
Others	7,721	5.9%^∗^	43.0%	29.9%	21.2%	20.4	23.5	30.9	40.0	29.1	Sig.<0.001
	455,491	5.2%	42.9%	30.9%	21.0%						

^∗^Percentage above the expected for this age (statistically significant). Sig. K-W test < 0.001 for all deviation. Growing LWD with age.

**Table 6:** Accidents and LWD by weekday (hour of the day; hour of work) and age in construction (Spain), 2011-2018.

	Accident number	% of the row				LWD (average)					K-W test
		-24	25-39	40-49	+50	-24	25-39	40-49	+50	Total	Sig.
Monday	113,259	5.2%	43.5%^∗^	30.8%	20.4%	21.47	26.43	31.80	38.51	30.30	Sig.<0.001
Tuesday	92,220	5.3%^∗^	43.6%^∗^	30.6%	20.5%	20.05	26.23	32.56	39.50	30.56	Sig.<0.001
Wednesday	87,214	5.4%^∗^	42.9%	30.8%	20.9%	21.87	26.90	33.14	40.32	31.36	Sig.<0.001
Thursday	77,644	5.0%	42.7%	30.8%	21.6%^∗^	23.44	28.58	35.43	42.75	33.49	Sig.<0.001
Friday	72,107	4.9%	41.0%	31.5%^∗^	22.6%^∗^	24.24	30.46	35.84	43.42	34.78	Sig.<0.001
Saturday	9,983	4.9%	45.9%^∗^	31.3%	18.0%	30.59	35.06	39.74	51.21	39.21	Sig.<0.001
Sunday	3,064	4.0%	45.2%^∗^	32.1%	18.7%	24.04	32.29	39.88	43.57	36.51	Sig.<0.001
Morning (8 to 14)	295,921	5.2%	43.1%	31.0%	20.8%	21.79	26.95	33.14	40.21	31.36	Sig.<0.001
Lunchtime (14 to 16)	31,489	4.9%	42.6%	31.3%	21.2%	23.35	30.47	35.70	43.93	34.61	Sig.<0.001
Afternoon (16 to 19)	92,480	5.2%	41.9%	30.7%	22.2%^∗^	22.19	28.54	34.20	40.88	32.69	Sig.<0.001
Others (19 to 8)	35,601	5.3%	44.9%^∗^	30.5%	19.3%	24.48	29.23	35.46	44.10	33.74	Sig.<0.001
First 4 hours of work	277,602	5.2%	43.3%^∗^	30.9%	20.6%	21.75	27.04	33.36	40.33	31.46	Sig.<0.001
More than 4 hours of work	177,889	5.1%	42.3%	30.9%	21.6%^∗^	22.89	28.74	34.26	41.74	32.96	Sig.<0.001
	455,491	5.2%	42.9%	30.9%	21.0%						

^∗^Percentage above the expected for this age (statistically significant). Sig. K-W test < 0.001 for all injured body parts. Growing LWD with age.

**Table 7:** Accidents and LWD by injured body part and age in construction (Spain), 2011-2018.

Chi square = 6860.867028		Accident number	% of the row				LWD (average)					K-W test
Sig.<0.001			-24	25-39	40-49	+50	-24	25-39	40-49	+50	Total	Sig.
Not specified		1,385	5.9%^∗^	44.2%^∗^	29.6%	20.3%	22.9	29.1	37.9	45.0	34.5	Sig.<0.001
Head	Eye(s)	27,138	5.5%^∗^	46.9%^∗^	30.4%	17.2%	9.0	9.2	11.0	12.2	10.2	Sig.<0.001
	Head (others)	13,016	5.1%	39.4%	30.4%	25.1%^∗^	17.7	22.5	25.8	34.2	26.2	Sig.<0.001
Neck		11,626	6.4%^∗^	55.8%^∗^	26.1%	11.8%	18.6	21.4	26.3	33.0	23.9	Sig.<0.001
Back		84,187	4.0%	44.2%^∗^	32.0%^∗^	19.8%	15.8	20.6	24.1	29.5	23.3	Sig.<0.001
Trunk		22,198	3.0%	36.1%	33.2%^∗^	27.8%^∗^	20.2	24.8	32.1	41.2	31.6	Sig.<0.001
Upper limbs	Shoulder and humerus joints	20,086	4.8%	36.7%	30.5%	27.9%^∗^	31.5	34.8	48.9	70.0	48.8	Sig.<0.001
	Arm, including ulna joint	25,046	4.2%	40.3%	33.3%^∗^	22.3%^∗^	24.9	35.7	43.7	51.1	41.3	Sig.<0.001
	Hand	31,490	6.9%^∗^	46.3%^∗^	28.6%	18.3%	22.0	25.8	29.8	33.5	28.1	Sig.<0.001
	Finger(s)	55,515	6.6%^∗^	44.6%^∗^	29.2%	19.6%	23.2	26.9	31.6	35.3	29.7	Sig.<0.001
	Wrist	16,435	6.9%^∗^	44.0%^∗^	29.8%	19.3%	28.2	35.1	44.4	50.2	40.3	Sig.<0.001
	Upper limbs (others)	5,481	4.1%	40.1%	32.0%^∗^	23.8%^∗^	24.1	33.8	42.7	51.4	40.5	Sig.<0.001
Lower limbs	Hip and hip joint	2,387	3.6%	35.4%	33.2%^∗^	27.9%^∗^	36.4	36.5	44.5	59.9	45.7	Sig.<0.001
	Leg, including knee	58,269	4.0%	38.1%	33.0%^∗^	24.9%^∗^	31.4	39.9	41.3	44.8	41.3	Sig.<0.001
	Ankle	29,639	6.9%^∗^	47.7%^∗^	29.4%	16.0%	19.9	27.5	35.1	41.7	31.5	Sig.<0.001
	Foot	28,712	6.5%^∗^	45.2%^∗^	29.5%	18.8%	22.1	30.0	39.4	45.7	35.2	Sig.<0.001
	Toes	3,853	5.6%^∗^	43.2%^∗^	30.4%	20.8%	23.9	30.6	35.7	38.4	33.4	Sig.<0.001
	Lower limbs (others)	6,620	3.5%	38.0%	33.1%^∗^	25.4%^∗^	30.2	39.9	45.9	51.2	44.4	Sig.<0.001
Multiple parts		12,408	4.1%	39.2%	31.5%^∗^	25.2%^∗^	32.9	46.7	57.2	66.2	54.3	Sig.<0.001
		455,491	5.2%	42.9%	30.9%	21.0%						

^∗^Percentage above the expected for this age (statistically significant). Sig. K-W test < 0.001 for all injured body parts. Growing LWD with age.

**Table 8:** Accidents and LWD by end cause of the leave and age in construction (Spain), 2011-2018.

Chi square = 1346.481187	Accident number	% of the row				LWD (average)					K-W test
Sig.<0.001		-24	25-39	40-49	+50	-24	25-39	40-49	+50	Total	Sig.
Unknown	31,238	4.5%	43.4%	30.5%	21.6%^∗^	33.4	35.1	39.4	44.5	38.4	Sig.<0.001
Recovery/healing	420,206	5.2%^∗^	43.0%^∗^	30.9%	20.8%	21.2	26.3	31.8	37.8	30.1	Sig.<0.001
Death	684	2.3%	23.8%	33.9%	39.9%^∗^	2.1	2.2	5.6	8.9	6.0	.032
Legal incapacity for work	2,619	1.1%	23.4%	30.9%	44.7%^∗^	218.4	276.7	274.5	261.9	268.8	Sig.<0.001
Absence	744	11.4%^∗^	52.3%^∗^	27.3%	9.0%	36.0	38.8	44.2	41.1	40.2	.735
	455,491	5.2%	42.9%	30.9%	21.0%						

^∗^Percentage above the expected for this age (statistically significant). Sig. K-W test < 0.001 for all end causes. Growing LWD with age.

## Data Availability

This is a secondary data analysis study using official accident data. The data for this analysis were collected from the official Workplace Incident Notification Forms, held on file at the Ministry of Labor, Migrations and Social Security in Spain. The ministry provides the data if they are required for research.
